# Supplementary material for: A role for cortical interneurons as adversarial discriminators
Source: PLoS Comput Biol. 2023 Sep 28;19(9):e1011484. doi: 10.1371/journal.pcbi.1011484 (PMC10538760; doi:10.1371/journal.pcbi.1011484)
Supplement: Attachment — Submitted filename: response to reviewers.docx [file pcbi.1011484.s001.docx]

To the reviewers and editors,

First, a sincere thank you to the reviewers for your helpful comments. We agree with many of these critiques, and in particular the response that this submission had a split identity. It was not quite a machine learning paper, and not quite a neuroscience paper, but somewhere in between.

Given our interests and those of the journal, we believe that this is most useful as a work of neuroscience theory. To this end we have significantly rewritten this manuscript. A new abstract and introduction more clearly indicate this theory’s relevance for neuroscience, and we would now like to re-title the manuscript, “A role for cortical interneurons as adversarial discriminators.” We think this better sets expectations as to the manuscript’s aims.

There are significant changes throughout the manuscript. For example, we reorganized the main justification of this work in the abstract and introduction. You’ll find that it now centers the need to identify the biological underpinnings of approximate inference, rather on the need for algorithms development. There is now a much clearer discussion of the biological plausibility, for example. We have also expanded the background about why approximate inference over a generative model is a good framework for neuroscience theory.

We have also created two new figures, and removed two figures. This paper presents a biological theory, and the purpose of all figures is to help evaluate the biological plausibility. This work is no longer attempting to establish a new machine learning algorithm that beats any benchmarks. For this reason, after careful consideration, we have decided to remove the CIFAR and moving MNIST figures, as these had little bearing upon the biological plausibility of the main proposal, nor helped to communicate the essence of the biological theory.

Considering the scope of changes requested, we hope the reviewers can re-evaluate this new manuscript with new eyes. In writing this paper we believe that there exists a role for a truly theoretical neuroscience, as in theoretical physics, that begins from first principles and makes predictions as to what can be found. This requires taking ideas from pure theory (for us machine learning) and presenting the best case as to how these ideas might map to biology and be tested experimentally. We thank the reviewers for their recognition of the importance of this genre of work.

Sincerely,

The authors

*Point-by-point response to comments:*

**Comments to the Authors:**

Reviewer #1: Summary
=======

The authors consider the problem of learning a generative model of data in a variational inference setting.
In contrast to other works, which often assume conditional independence of neurons in each layer of the generator, they consider networks in which each layer can be recurrent.
They argue that since biological networks are recurrent, this is a more natural setting for (generative) models of brain computation.
To solve the computational issue of optimizing a generative model in the presence of recurrence, and to make learning more compatible with biophysical constraints, they hypothesize that additional "discriminator" neurons in each layer together with alternating bottom-up and top-down drive on the generative neurons implement an adversarial learning mechanism.
They demonstrate with a toy tasks, and also using moderately challenging machine learning datasets that their suggested principle can successfully learn generative models in various network architectures also in the presence of recurrence.

The manuscript is technically sound, the authors clearly understand the mathematical and computational principles underlying (amortized) variational inference and adversarial learning.
The presentation is moderately clear, but should be improved (see detailed comments); most importantly the methods should be significantly extended (or and appendix should be added) with derivations and details on the formal setup of the different tasks (e.g., the graphical models).

My main concern is that I could not clearly understand what novel insights the authors would like to communicate (this issue is potentially already highlighted by the, in my opinion, rather vague title). Is this a machine learning paper that has important implications for biology? Is this a biology paper heavily inspired by machine learning? If the main contribution is in terms of algorithms (large parts of the introduction and results read like it is), then I believe paper is severely lacking a discussion of the existing literature and comparison to existing models; a brief Google scholar search reveals works from 2016 which already consider adversarial learning with recurrent networks. If the main contribution is on the biology side, then I would expect significantly more discussion of the challenges of implementing the model in biological substrates (to justify the authors claim "This extension of the Wake/Sleep algorithm offers a bridge between the computational objective of Bayesian inference and the biological implementation in synaptic plasticity."). Specifically, from a biological perspective I see the following issues:
- What would a "learning rule" (which the authors mention) look like according to this framework and how would it be implemented in a biological substrate?
- requiring a precise, distributed timing (oscillation algorithm)
- relying on backprop (through time!)
- using LSTMs
- requiring careful (hyperparamter) tuning to overcome instabilities of adversarial learning
- using divisive normalization
- using ADAM
- using convolutions
The simulations the authors provide are all rather abstract (wrt biology) and seem to assume that all of these complications are somehow solved in the biological substrate.
From a methodological perspective, evaluating the quality of the learned generative model is mainly left to the reader via visual inspection; I think the manuscript would benefit from more quantitative evaluation measures.

If I may be so bold to make a suggestion: it seems to me that the main contribution of this manuscript lies in the extension of wake-sleep to non-Gaussian, non-factorizing distributions using adversarial learning. If novel (please check!!!), this is an important contribution to the statistical learning literature. In this case, I can envision a clear, compelling manuscript focused on this topic which could be published at appropriate high-impact (ML) venues. In an additional (perhaps "perspective"?) manuscript the authors could then highlight the implications of this new machine learning model for learning in biological substrates.

Please receive additional notes for the authors below.

Your main concern was well-taken and kept at top of mind as we re-wrote this manuscript. Hopefully you’ll find the aims and take-away much clearer.

The work now does not position itself as a novel algorithm. Instead, as theoretical neuroscience, the primary goal is clear and even-handed evaluation of the plausibility of our adversarial hypothesis proposal in the brain. As such the primary consideration for the merit of this manuscript is likely the thoroughness of this evaluation. For this reason we have greatly expanded it. There is now a discussion of the learning rules required, for example, and possible solutions to the issue of credit assignment.

Not all aspects of the simulations were meant as embodiments of our biological hypothesis. We understand this narrow scope was not made clear, and we now provide text about our philosophy of modeling and what should be taken away. Neural networks require commitments to smaller details than can be known about the brain, but we believe such experiments are still valuable sources of intuition.

General notes
=============
- motivation rests on prediction in time, but model does prediction "in space"; please keep the two separate; how can you motivate the latter?

We believe this was us being imprecise about our use of ‘prediction’, which we had actually used in both senses in the manuscript but failed to delineate. We now avoid the use of ‘prediction’ unless talking about prediction in time. It should be clearer now.

Our main motivation, as with other representation learning models of the brain, is the generative modeling “in space” of neural populations. We now spend a little more time describing this objective and linking to previous literature.

- why are learning a generative model and inference in it meaningful objectives for the brain to solve? first, a generative model does _not_ allow prediction of _future_ inputs (in particular not the model considered here); second, why transform sensory input into a "high-level" representation; all computation implies non-increasing information, so what would the brain try to achieve?

Hopefully our revision is now clearer on this point. While we think it should be able to stand alone in this respect, we can provide a direct response here as well.

Generative modeling and inference are meaningful objectives because they make precise the two subproblems of forming representations of the world. Take the example of object categorization. (In this case, an object category = the representation.) Because sensory information is ambiguous, the brain should guess categories probabilistically, with uncertainty. This is of course just probabilistic inference: given inputs x and categories c, the brain wants to estimate p(c|x).

Next, we can state that our categories ought to be as good as possible. As you state, it's true that forming representations involves throwing away information about the world. But at least we can learn categories that throw away as little information as possible – and in fact, the categories that best preserve information are those implied by the best generative model of the world given categories. This is the justification of this framework that is presented in the Wake/Sleep paper by Hinton, Dayan, etc., in the info-theoretic language that the goal is to build compressed code for the world. (Briefly, the connection between generative success and information is that the generative model implies a distribution over x, p’(x)=E_c [p(c|x)], and the information lost when keeping only categories is the KL divergence between this p’(x) and the true data distribution p(x).) So learning good representations in this information-theoretic sense is the same as learning a good generative model.

Without saying what these representations are in the brain, exactly (are they categories? Object boundaries? Colors? … ) probabilistic representation learning still gives us a framework for thinking about learning. Namely, that the representations should still preserve information and that inference should be optimal. There are other justifications besides this information-theoretic one, such as self-consistency. One may also take representations and generative models for granted, and ask how they might be learned; this is the approach of Kingma and Welling’s VAE paper, for example.

- much of "Comparing phases of an oscillation" (ll278-319) is not really results and would maybe better suited for the discussion

We’ve moved this text to the discussion.

- how does the discriminator gate plasticity biologically?

This is now described in a dedicated section in Results.

Line notes
==========

Many of these line notes are now longer relevant as much of the paper has been rewritten. Nevertheless these were important in the rewrite. We respond by point where as specific question was posed.

- ll35-36: or they just compare new experience with old experience? or they just learn not to be surprised? a lot of (implicit) assumptions in the first sentence! if not explained further at least add references

This paragraph is now expanded with further citations. The goal of this paragraph is now mainly to establish the extent to which many fields believe the brain has a generative model.
- l38: /if/ the previous hypotheses are correct, then learning adapts the predictive model

We have also removed ‘predictive’ unless talking about future information, per your comment below.
- l40: what kind of benefits?

These are now listed by subfield.

- l42: "...predict must..."? merge issue?
- ll42-46: kind of complicated way to explain (Bayesian) inference; simplify?
- l57: "abstract"/"concrete" in what sense?
- l69: what is an internal model? a generative network? a predictive network? it would be useful to clearly distinguish the two (the former transforms high-level representations to low-level activities, the latter predicts future high-level representations from past high-level representations)

Thank you for this; we now only refer to predictions when referring to time. Previously we used these interchangeably.

- ll76-79: motivation is quite weak ("recurrent connections exist, hence correlations expected"); first, there _are_ definitely correlations (see any paper on neural correlations) and second, one could argue from a functional perspective: models that capture additional correlations are more powerful; can you give an example where this additional computational power would be useful?

The introduction is now motivated rather differently. Where we bring up this issue later, we make it clear that this is about the plausibility of competing models for approximate inference in the brain, almost all of which are “likelihood-based” and require evaluating likelihoods, which we argue is not plausible (due to recurrence and the resulting correlations.)

- l82: "incoming synapses" from where? top-down? recurrent? would be helpful to be explicit here
- ll87-95: could you briefly summarize the "justification" you allude to? could you provide an intuition for why such adversarial learning could be useful?

Much of the algorithmic justification is now in the Results, and expanded upon there.

- l93: who is "they"?

No longer present; the referent was ‘connections’
- l101: "...share the By contrast..."; merge issue?
- l109: "consequences"
- l110: "their similarity to known results"; sounds a bit weak, maybe formulate stronger if you are talking about experimental results? for example "explain empirical findings"?
- ll124-125: using q for both the true data distribution and the approximate posterior is quite confusing; is it ever possible to match the true distribution in a finite-data scenario?

The use of q is convenient as it refers to all “wake”-phase distributions (see comment immediately below).

It is vanishingly unlikely to match the true distribution exactly, for two reasons. Even if real-world data has the same functional form as the generative model, the bias and variance of an estimator only decreases asymptotically with increasing data. Secondly, the functional form of real-world data is unlikely to ever precisely match any generative model’s assumption. This makes it effectively impossible to have zero error in matching a true generative distribution.

- l134: how is q(x,z) defined? is it the combination of the approximate posterior with the true data distribution as a prior?

Precisely, q(x,z)=q(z|x)q(x); this prompted the use of the q(x) notation. This is now hopefully clearer in the text.
- l134: explain a bit more why matching the joint distributions fulfill the two objectives, i find it non-obvious (e.g., it is a necessary or sufficient condition? how to arrive from matching joint distributions at the two original objectives?)

This should be clearer now. It is necessary and sufficient; q(x,z)=p(x,z) if and only if both q(x)=p(x) and q(z|x)= p(z|x). In the right direction, the marginals must match if the distributions match, as p(x)=sum_z p(z,x)= sum_z q(z,x)=q(x) The left direction follows from the definition q(x,z)=q(z|x)q(x) and p(x,z)=p(z|x)p(x).
- l143: i don't understand: also VI matches distributions, so why use the joints here?

VI is also a strategy of matching these joint distributions. Our point was that these objectives are the same. But whereas doing gradient descent on the ELBO (free energy) requires estimating a likelihood, an adversarial method only needs samples.

- l152: what is a transport problem? so far you have only stated that p(activity neuron i) != p(activity neuron i|activity of all [other] neurons); also, maybe don't assume everyone is familiar with the weight transport problem

This paragraph no longer appears as such. Issues around this dependencies have been restructured to surround Figure 2, the toy example.

- ll155-165: if possible (according to journal), some math would really help to get the point across, and maybe a figure

This has been reorganized and now appears only in association with figure 2.
- l175: "one solution" ;)
- l182: define "easily"; how are LSTMs implemented in the brain? how is backprop through time solved?

Very true: we only meant that the overall adversarial framework can be extended to time-varying inputs, not that all time-based algorithms are easily implemented in the brain.

- l186: it should probably read something like "two distribution are better aligned if a discriminator can not tell samples from them apart"
- l215: and Deperrois et al.

Indeed!
- l221ff: you mention learning rules, but only show objectives; what would the learning rules look like? what quantities are used in the learning rules and how do they get there, i.e., are they local? who teaches the discriminator? what is the architecture of the discriminator? does learning in your model need backprop (if so, how do you solve all the issues associated with bio bp?)? do you need the regularizer? if so how is it implemented in bio?

These are very important questions, and we now dedicate a lot of space to answering these. We hope the new manuscript satisfies these concerns.

- l243: how correlated are these increases/decreases in synaptic strength with the source neuron type? i guess your model would predict opposite changes for projections from exc/inh neurons? is there evidence for this?

Unfortunately this data is so far known only without reference to cell type. The predictions for learning rules are now given explicitly as equations. It turns out that the plasticity for projections from E/I onto the discriminator cells actually do not depend on cell type, only presynaptic activity.

- l249: i'm a bit concerned about the timescale here: if oscillations have periods of ~10ms, and the neurons response time is also on the order of 1-10ms, doesn't the somatic output "mix up" information from the two phases? to me it seems like you would need longer timescales if you want to separate the two phases and want to take the biophysical dynamics of neurons into account

Very true; this claim is directly from Honey et al. Perhaps such comparisons of an internal model are more plausible at slower, alpha or beta, frequencies. We have removed this explicit reference of timescale.

- l255: maybe mention here explicitly that this a difference to the "standard" approach; in your case, the joint distribution of a layer _can not_ (trivially) be further decomposed into a product of distributions over neurons and that you can thereby capture arbitrary (conditional) statistical dependencies between neurons in one layer!

Thank you, this was done.
- l264ff: an important difference to wake sleep is the fact that there's is a discriminator, right? also not sure what "the above setting" and "this algorithm" refer to

Yes, this is similar to the original Wake/Sleep algorithm in its objective, except we do this adversarially. This sentence was unclear, and it is has now been replaced.

- l270 (eq 5): could you provide a derviation of this objective from eq 2; it doesn't seem completely obvious over which distributions the expectations should be taken; for example, didn't you assume a perfect inference model and shouldn't the first expectation of the second term be over the join q(z{i+1}, x)?

Yes, thank you, this first expectation should be of that form. This derivation actually begins from the equation directly before. We are replacing KL(q(z_i|z_{i-1}) || p(z_i | z_{i+1})) with an adversarial alignment of those two distributions over z_i. This should be more clear in the text now.

- l280ff: very cool interpretation of oscillations!
- l285ff: it seems to me that two things are mixed up here; first, oscillations switch non-discriminator neurons from top-down to bottom-up drive and provide a teaching signal to the discriminator(?); and second the activity of the discriminator itself is interpreted as the oscillation; the oscillation can hardly be teaching the discriminator and be generated by it simultaneously, can it? i think this needs to be explained better

This section has now been moved to the discussion, and edited for clarity.
- l308f: which plasticity switches sign? that on "inference/generative network" neurons or those on discriminator neurons?

Both switch sign (given the same activity patterns).
- l321: which algorithms? it's not clear that the two previous sections describe two different algorithms; rather to me it seems like wake-sleep + discriminator describes an abstract ML model and the oscillation part describes a potential biologically plausible implementation

Yes, this section is unclear. We have removed this and added mention in the discussion that the brain may learn with an adversarial algorithm on both timescales, discriminating between wake and sleep, and oscillatory phases. Both are meant as biological hypotheses.
- l324: which objectives? (see previous comment)
- l334ff: i'm quite confused: i would've expected that learning adjust connections in the internal model (T->A,B) rather than in the external drive (A->B,C)!? here it seems like the internal model doesn't learn anything but rather the representation is fixed and the stimulus is somehow mapped to this representation; the logic behind the setup should be explained a bit more i think; in addition, aren't the results obvious: if you assume independence where there isn't, you learn worse?

Yes; we have updated the figure to be more consistent with terminology. T is the external ‘teacher’ and A is the internal model.

This is indeed a trivial / obvious model. And yet, a great many papers ignore this issue, and many computational neuroscientists believe that prediction is all you need to minimize free-energy. This is wrong due to this recurrence. Since this belief is so widespread we thought it necessary to construct a minimal example.

- l362: what does "during recurrence mean"? do you run the network until it reaches a steady state? for Xms? one "step"?

This was meant to be 1 step. The text now removes this ambiguity.
- l375ff: adversarial learning in recurrent architectures was done before in ML, maybe mention this; do they also find that it doesn't work very well for inference?

To our knowledge no paper has attempted adversarially-learned *inference* using a recurrent inference model. As you mention, many have combined GANs with recurrent networks, though the purpose in these papers is image generation and not representation learning.

- l383: "biology may have found solutions to stabilize this approach" is a bold claim and would benefit from support (futher arguments why this should be the case or references)

This was admittedly a speculative sentence. We have converted this into a more balanced discussion of the fragility of adversarial algorithms in the Discussion
-l393: "signifying that the inference network maps to the support of the generative posterior" seems like a bold statement given these results; your objective now explicitly contains a reconstruction loss, so it is not surprising that reconstructions become better; still latent representations of data could reside in a different part of the latent space than what you use for spontaneous generation; more analysis needed if you want to make a strong statement here; also, would be good to quantify the generation/reconstruction quality, maybe FID?

True, this is hard to substantiate. We have removed this strong statement, but wish to mention, in our defense, that if the inferred representation for a digit maps (through the generative model) back to the same digit, the representation must be in a region of high density in the generative posterior. However, this is only a point estimate, and for this reason we do not believe we can rigorously support this statement.

This relates to a different issue, referenced immediately below, about quantitative evaluation. Although FID is a possible measure for generative quality (although it is still problematic in various aspects), this is not a measure of the quality of approximate inference, which is also a main aim.

- l400: even with Berkes' approach you rely on visual inspection of the plots; could you distill it into a single number that could be compared between algorithms?

This is also very difficult. The same factors that make building approximate inference algorithms hard for recurrent models (namely, that calculating the likelihood is hard) makes it very difficult to quantitatively measure the quality of approximate inference. If such a measure existed, one could do backpropagation and optimize towards it.

In principle, one could summarize the Berkes’s histogram approach into a single number: the expected distance of all points from the y=x axis. (This is related to the KL divergence on binned histograms). Yet, this seems to remove information that is otherwise available in the plot. We strongly feel that this scalar-value quantification is not necessary for the overall evaluation of the plausibility of the biological hypothesis.

At high level, however, we acknowledge the need for a comparison to the performance of other algorithms. For this reason we now include the performance of a variational approach to the same problem of approximate inference over this recurrent generative model, using the same recurrent recognition model. Since this model is outside of the class of networks for which one can easily write the generative likelihood, this algorithm performs terribly.

- l408: how would divisive normalization be implemented? with an additional population? how about error transport, locality of information etc?

We chose divisive normalization because it has a long history, e.g. the referenced Heeger et al.

Yet, as the below comment mentions, this additional experiment arguably adds little to this work. For this reason we have removed this experiment.

- l414: wasn't this explored in the ML literature before? is it surprising that it works/what is the novelty here? how bioplausible is this setup?

The original point of this figure was to demonstrate that hierarchical inference is possible. But this arguably adds little above the work in the machine learning literature, such as Larsen et al. (2016), Huang et al. (2017), and Belghazi et al. (2018), which demonstrates the same. After careful consideration, and an honest discussion about what is learned in each plot, we have decided to remove this figure. This work is no longer attempting to establish a new machine learning algorithm that beats any benchmarks. It is a biological theory, and any figures that suggest the contrary are likely to be distracting from the intended main takeaways. The removal of this figure makes more room and emphasis for our biological theory.

- l419: interesting point about delays but first, this should come way earlier, and second this should be discussed more
- l429: non-Gaussian or non-diagonal or both?
- l430: how bioplausible is any of this?

We have added a discussion of this. It should be more bioplausible than the rest, as it is a minor change to the algorithm with the exception that it now acknowledges the passage of time.

- l448: "It is general [with regard to]"?.
- l457: is this objective really easier? in hierarchical networks gradient descent on the variational free energy doesn't seem much more complicated than your proposal; if you want to make this point, explain explicitly why yours is easier; indeed just thinking about the specific timing requirements of switching discriminator targets in a continuous-time system like across cortex seems daunting

Yes, we think! When recurrence exists it becomes extremely difficult to evaluate the likelihood of a network state. Because of neuronal correlations, calculating the likelihood requires observing the entire network state, and the ‘observer’ (wherever that is) needs to have a perfect model of likelihood. It’s only by ignoring recurrence that it becomes feasible, since then at least the objective is a sum of local prediction errors. But this is unlikely to be possible in the brain.
- l467: in Deperrois et al., plasticity in wake is also training the encoder (in addition to the discriminator)

Ah, sorry for this, yes. Both the encoder and discriminator learn in wake.

- l477: what does "classify oscillations" mean?

“discriminate” objectives; to act as a discriminator that aligns activity during phases of oscillation.

- l483: is it necessary that the plasticity switches sign? couldn't you also just switch the target? i assume the theory suggests the latter; if so, are the two equivalent?

Assuming that by ‘target’ you mean the goal of learning, which is increasing/decreasing the discriminator’s activity, then yes, these are equivalent. Switching the sign of an otherwise identical plasticity is the same as performing e.g. gradient descent on a negative of the original objective.

- ll490-498: this is a rather general prediction from learning/using generative models during sleep, right? it doesn't seem specific to your hypothesis

Yes, this is true. But it is surprising this hasn’t been done! So many theorists care about generative modeling. Any interested experimentalists had better start here, we think, and then continue on with the experiments testing the adversarial hypothesis.

- ll499-504: cool experiment! seems extremely challenging, and I'm not sure it's obvious why this would be the case; maybe expand a bit (encoder tries to invert generator)?

Indeed, this would be difficult, but it is feasible with current technology. We’ve added a few more details as to what this would require experimentally.

- ll505-512: not sure i understand; maybe chat with an experimentalists to see whether its clear what you are suggesting?

Indeed, this is the result of such conversations. These experiments would actually not be overly difficult. We have expanded this section to make it clearer what we are proposing.

- ll514-517: yes! maybe this point could already be picked up earlier in the manuscript; i feel its essential

Yes, it seems that saving Limitations for last does not work well. We have moved these into the relevant sections in Results
- l526ff: i think this should also be mentioned earlier; i was wondering in the results quite a bit how you optimize, in particular because you mention "plasticity rules"
- l532: maybe end the manuscript on a stronger note?
- l564: explain your implementation of recurrence more
- l592: not mandatory, but i prefer all details in the manuscript; if everything is in one place it's easier to look it up and one doesn't need to assume the code is always available

We have added many more details to Methods.

Figure notes
============
- figure 1: maybe keep all recurrent connection of the network in "our hypothesis" in addition to discriminator connections; not sure what "target neurons" are; do you just mean "neurons"?

This figure is no longer present.
- figure 2: is b the same as the first panel in c? maybe only keep one? in e, why is the adversarial error initially so small? should both errors start at the same point?

Yes, good point - the adversarial and single-neuron versions were initialized randomly, and thus had different random starting errors. As this makes a comparison hard, we have changed this such that both algorithms begin with the same random initialization. We have also changed this to show the learning trajectory of the adversarial algorithm over 5 random seeds .

- figure 3: in a, not sure what the "combine" operation is, please explain; in b,c please explain the three different tasks (reconstruction, denoising, spont. acivity); in c, why are there three arrows into the discriminator, what is discriminated against what? possible to indicate in figure?

A better word would be “interpolate”. We have added a more detailed caption with the answers to these questions.

- figure 4: why reconstruct from z1? isn't that rather easy? how about reconstructions from the deeper layers? are these good reconstructions? are these good generated images? provide at least some baseline to compare to (plain autoencoder? wake/sleep?) and some quantitative evaluation of the images (FID?)
- figure 5: c, explain the different tasks, and again, are these results good or bad?

=========

Reviewer #2: In this manuscript, Benjamin and Körding propose a Wasserstein GAN model for inference in recurrent cortical circuits. Their model proposes alternating "wake" and "sleep" phases, in which the discriminator is alternately driven by bottom-up inputs and top-down predictions. They then argue that this algorithm bears some similarity to cortical learning phenomena, and provide proof-of-principle simulations to demonstrate its efficacy. I find the broad idea of the proposed algorithm somewhat interesting. However, I think the results of the present manuscript fall short of what is promised---namely, "the missing link between a widely hypothesized computational goal of learning and its implementation in neural systems"---and I do not believe it is suitable for publication in its present form.

Below, I give more detailed comments, roughly separated by importance but not ordered within each category. I would like to preface my remarks with the caveat that my knowledge the literature on sleep or on cortical oscillations is superficial at best, so I cannot offer useful comments on those aspects of the paper.


Major comments:

1. The algorithmic challenge the authors aim to address is the complicated dependency structure induced by recurrent connectivity. However, I found the presentation of their proposed solution hard to follow on first reading. I would suggest that the authors separate the mathematical construction of their model from the discussion of its possible biological implementation. Concretely, I would propose the following organization: (i.) introduction of the problem of recurrence and of the proposed WGAN algorithm at an abstract, mathematical level; (ii). proof-of-concept simulations to demonstrate that the proposal is an effective solution; (iii). mapping onto biology, existing evidence, and proposed experimental tests. Moreover, to allow the reader to understand precisely what is being proposed, I suggest that the authors provide pseudocode for their algorithms (as in, for example, the original WGAN paper by Arjovsky, Chintala, and Bottou). This could be included in a box. The concrete problem addressed by the paper should also be stated precisely in the abstract, rather than with vague generalities. I believe this reorganization would improve the readability of the paper, and make it easier to evaluate its contributions.

Thank you – we have now significantly reorganized the manuscript in response. Our main concern, as brought up by both reviewers, was the lack of clarity around the specific aims of the paper. The reorganization reflects a greater emphasis on providing a theory for the brain, and a lesser emphasis on providing a novel algorithm for the ML community. We cannot mention of our changes in this short response; we hope you may re-read this manuscript with fresh eyes, and that it now reads more clearly.

2. The authors mention the fact that the discriminator in (2) requires access to the full network state, which is not biologically plausible. They do not discuss in detail the fact that the algorithm in (3-5) shares similar limitations: depending on the size of the clusters in (3), each local discriminator must receive input from a potentially very large population. Therefore, the efficacy of their approach would seem to rely on each layer of neurons being relatively small. For their algorithm to be a compelling proposal, the authors must demonstrate that this is not an unreasonable requirement. Namely, how large might one expect the clusters of neurons implied by Figure 1 to be? Though doing so is beyond the scope of the present manuscript, another requirement would be to show that biologically-constrained architectures can successfully be trained on adversarial tasks, given the known instabilities in GAN training (as discussed, for instance, in the paper by Donahue and Simonyan that the authors themselves cite). This is likely a particularly strong limitation if the layers are large. With this in mind, I think it is also important for the authors to discuss the plausibility of stable biologically-plausible WGAN training in greater depth than the cursory mention in Lines 526-532.

We have now added a much larger section about biological implementation, addressing this and several other issues. Our goal is not to persuade the reader that this is definitely implemented by the brain, but rather present hypothesis with all known arguments for and against it. To that end the concern about dimensionality is now raised to a top-level concern and mentioned in the abstract. On the issue of the size of the local populations matched by the oscillatory algorithm, we now emphasize that this must be a few tens of thousands at most, on the order of a cortical columns, writing, “for computational feasibility, the size of these matched populations must be on the order of tens of thousands of neurons”

3. Based on my reading of the paragraph starting on Line 236, it seems the authors propose that the discriminator is trained online to maximize the WGAN objective, with updates performed after each individual sample is received during both phases, which are separated in time. Is this an accurate understanding of the proposal? If so, the authors should demonstrate that this approach actually succeeds, as it appears from the methods that their experiments were performed with batch sizes much greater than one. If not, the authors should discuss how the information required to perform batched updates might be stored.

This is indeed the proposal, when the timescale of comparison is on the order of oscillations However, we disagree that repeating such experiments with a batch size of 1 is necessary as, algorithmically, we already demonstrate that a single-sample comparison succeeds. The ontological comparison is at the algorithmic level. The batch size, on the other hand, is merely an arbitrary computational implementation for digital efficiency. The effect of batching updates across many samples is merely to decrease the noise due sample subsampling (which can furthermore be compensated for by a larger learning rate).

This concern relates to a larger discussion about the role of these neural network simulations in our paper. We have now added a paragraph before these models that describes this more clearly. This paragraph is as follows:
“In this section we present various neural network simulations to demonstrate the capabilities of an adversarial approach, including both advantages and disadvantages. Our approach is not to include all possible biological details, or to claim that each computational component in these models has a direct corresponding element in the brain. Artificial neuronal network models differ from biology in many aspects. These `neurons' do not spike and have no dendritic trees, ion channels, or cell types. We assume that biologically plausible credit assignment is solved and use backpropagation where applicable. We also do not explicitly model any neuromodulators that might mediate the switch between wake and sleep. Finally, we make several arbitrary choices about the batch size, learning rate, and optimizer that are not meant as hypotheses about biology. In these and in other aspects our models should not be interpreted as earnest simulations of the brain. Rather, each experiment proves a general capability (or failure) of the algorithm which together can help adjudicate whether it is powerful enough for sensory learning in the brain.”

4. In the abstract, introduction, and Figure 1, much is made of the idea that "this manuscript assigns the role of discriminator to particular cell types distributed in the neocortex" (Lines 101-102). It would therefore be helpful to organize the proposed experiments based on which are tests of features particular to the proposed model rather than tests of features shared by related models (or of any model with phase-dependent plasticity). Moreover, the proposed experiments do not appear to address a crucial component of the alternating-phase model: that the connectivity of these interneurons should be determined by the conditional dependence structure of their synaptic partners. Naïvely, this seems challenging to probe. The authors should also elaborate on the question of whether the degree of connectivity required for an effective discriminator neuron is plausible.

Thank you, we have made this change, and also added a figure about these predictions. We still feel that these more general experiments are important to mention, as they have not been done and would need to come first in an experimental research program. Demonstrating a mechanism for distribution-matching in the brain is not possible without demonstrating this capability exists in the first place.

We agree that it would be very hard to verify that the connectivity of these interneurons depends on the conditional dependences of their targets is hard to measure, if not impossible given current technology. This would seem to require a detailed wiring diagram of mammalian cortex aligned to cell type information, which is not currently available. We have now added this as an explicit prediction in the discussion.

5. I do not find the numerical experiments to be well-motivated. Very little justification is given for the widely variable selection of tasks, types of recurrence, and network architectures. As the stated objective of the paper is to address questions relevant to biology, I believe it is important to give at least one proof-of-principle example of an architecture with greater biological motivation. At the very least, the authors should discuss what each experiment reveals, with reference to the paper's overarching goal.

Thank you, this was a major concern in our rewrite. We agree that these experiments were not specifically motivated. In the revision, we are much clearer about what each experiment demonstrates.


Minor comments

1. The clarity of the figures could be improved significantly. All of the diagrams and illustrations could be made much larger---the labels are currently so small as to be effectively illegible without zooming into the PDF---and additional labels could be added. For example, in Figure 3B, I presume from context that the top rows of the "Reconstruction" and "Denoising" subpanels show input images; this should be explicitly marked.

We have now reorganized the figures to make the text and information larger.

2. The proposed method for comparing phases of an ascending oscillation seems to resemble at a high level the idea of a stacked GAN, as studied by Huang et al., CVPR 2017 (arXiv:1612.04357). Provided that my understanding is accurate, this and other subsequent works from the machine learning literature should be cited, and the algorithmic (not implementational or interpretational) relationship to the stacked GAN should be highlighted. At the very least, the authors have argued for this setup as a way to tackle networks with recurrence, but I'm not sure how surprised one should be that such a thing would work given the existing literature on GANs.

Thank you for this reference, which we have added. This is broadly similar, and only difference being that the Stacked GAN works with a pretrained encoder while we learn an encoder from scratch.

3. The submitted manuscript is peppered with non-grammatical sentences and distracting typos. To give just two examples:

Lines 101-102: "These proposals share the By contrast, this manuscript assigns the role of discriminator to particular cell types distributed in the neocortex"

Lines 310-311: "In the hippocampus, for example, transitions between potentiation and depression and have been observed with phases of the theta rhythm in hippocampus"

4. In Lines 382-383, the authors write "We emphasize, however, that biology may have found solutions to stabilize this approach" in an effort to excuse the failure of their algorithm to successfully reconstruct MNIST digits. This is neither a necessary comment, nor a satisfactory one. As the network in question features many non-biological features, why appeal to biology here? If they are concerned that this somehow reveals a more fundamental deficiency of their model, this point should be elaborated.

Agreed; this was a cursory point when a larger discussion was merited. Such plausibility now has a greater role throughout the manuscript.

5. The authors mention, but do not discuss in detail, the WGAN algorithm's requirement that the discriminator is Lipschitz. At face value, this constraint would seem to affect the biological plausibility of their proposal, and warrants further discussion.

We have added a section about this issue in the broader section introducing the biological hypothesis. The Lipschitz constraint is plausible in that it depends 1) on the synaptic weights onto discriminator cells being bounded, which is already known to be the case biologically, and 2) the discriminator’s nonlinearity being not overly sharp. We have also added a section that describes what the discriminator’s cells learning rules would look like beyond the WGAN objective, such as in the original GAN objective.

6. Several papers published in computer science conferences---notably including the WGAN paper, published in ICML 2017---are cited only as arXiv preprints. This should be rectified.

Thank you; we have manually searched through our citations and corrected instances where this reference is outdated.
