# Supplementary material for: A role for cortical interneurons as adversarial discriminators
Source: PLoS Comput Biol. 2023 Sep 28;19(9):e1011484. doi: 10.1371/journal.pcbi.1011484 (PMC10538760; doi:10.1371/journal.pcbi.1011484)
Supplement: Attachment — Submitted filename: second response to reviewers.docx [file pcbi.1011484.s002.docx]

Dear reviewers and editors,

We wish to sincerely thank the reviewers for their thorough reading and very helpful comments. We believe this manuscript has been deeply improved by this review process. We hope the current changes have addressed the outstanding concerns.

We have worked to increase the clarity of key sections, and added paragraphs to describe in further detail the considerations mentioned. Of note is a section on Lipschitz constraints, and sections comparing this work in greater detail with the works of Belghazi et al, Deperrois et al., and Gershman.

Thank you again for this review process and your time throughout this process.
Sincerely,

The authors

_________

Two reviewers pointed out several points to improve the clarity of the manuscript, including the section on discussing Wasserstein GAN objective and the Lipschitz constraint. Of importance, is a more detailed comparison with the alternative proposals from Deperrois et al. and Gershman and well as to the method proposed in Belghazi et al.. Commenting on possible experiments that could enable falsification of different theories would be a great strength.

Reviewer's Responses to Questions

**Comments to the Authors:
Please note here if the review is uploaded as an attachment.**

Reviewer #2: I thank the authors for their effort to address my comments and those of the first referee. I believe the manuscript has substantially improved, and I am now generally in favor of its acceptance as an interesting theoretical proposal.

I have one suggestion, which the authors may consider in preparing the final version of their manuscript: In their discussion of the W-GAN's Lipschitz constraint in Lines 326-333, the authors briefly mention that having bounded synaptic weights is "aligned with natural biological constraints." I think this point is worth expanding upon, as the original W-GAN paper by Arjovsky, Chintala, and Bottou makes the case that "[w]eight clipping is a clearly terrible way to enforce a Lipschitz constraint," which would ideally be replaced with another approach. It would be useful to discuss in greater detail the idea weight clipping is not only biologically reasonable but also potentially (at least to some degree) imposed by biology, ideally with more citations to relevant experimental work.

Thanks for this great suggestion. We have added such details. And as you point out, while physical constraints resemble weight clipping, the brain could likely do better via weight penalties. We have considered this and have added the following text:
*“Biological neurons might achieve these properties via multiple strategies. Natural physical constraints upon the size of a synapse, as well as the achievable receptor density, represent an upper bound on synaptic strength that, if plasticity is otherwise unaffected, would resemble a simple weight clipping procedure. Surveys of synaptic volumes reconstructed via electron microscopy indeed find an upper limit of synaptic size (Bartol Jr et al., 2015). Alternatively, this constraint could present as a penalty (or regularization term) upon synaptic size, akin to the gradient penalty of Gulrajani et al. (2017). The effect of this penalty would be an inverse dependence of induced LTP upon the initial synapse strength. Indeed, classic plasticity induction experiments in cortical and hippocampus slices report such a dependence in excitatory cells (Bi & Poo, 1998; Liao et al., 1992; Montgomery et al., 2001; Sjostrom et al., 2001), demonstrating its plausibility in a potential discriminatory cell. The Lipschitz constraint required in a Wasserstein GAN thus naturally aligns with biological constraints and penalties upon synaptic size and efficacy.”*

A few other minor points:
1. In the discussion of the sampling hypothesis (paragraph starting in Line 214), it would be useful to cite Echeveste et al., Nature Neuroscience 2020, and also the original paper by Hoyer and Hyvärinen (NeurIPS 2002).

Thank you for this help in due diligence. We’ve added these papers to this paragraph.

2. In the first sentence of the abstract, are the words "throughout development" necessary?

No, this modifier is not strictly necessary; we had wanted to doubly emphasize the experience-dependent aspect of sensory learning. Since ‘development’ carries additional connotations we have replaced this phrase with “from experience”:
*“The brain learns its representations of sensory information from experience, but the algorithms by which it does so remain unknown.”*

Reviewer #3: This is a nice paper exploring the idea that the brain uses adversarial learning techniques to build a statistical model of the world. The paper argues that adversarial learning has a number of appealing aspects that make it a more plausible theory than competing theories based on variational inference. First, recurrent architectures can be handled seamlessly. Second, hierarchical models with local recurrence can be learned with local discriminators. This makes it easier to satisfy the constraints that are known from the architecture of the cortex. The authors make a high-level connection to oscillatory codes to justify a fast alternation between bottom-up and top-down modes, needed to learn the model.

The hypotheses advanced by the paper are interesting and in my opinion sufficiently distinct from prior work by Deperrois et al. and Gershman to warrant publication. There are however certain aspects of the paper that I think could be improved.

My main issues with this paper revolve around clarity. The text mixes and rapidly alternates between discussions of prior machine learning work, biological modeling and plausibility considerations. I understand the nature of the work requires plenty of discussion. But, despite the improvements, there's still too much context switching in the present version. I think that one more iteration of paper reorganization (rather than a rewrite) would be sufficient to improve readability.

We wish to thank this reviewer for their helpful comments. In response, we have aimed to improve clarity and in particular have made efforts to further separate the computational and biological sections. For instance, a full paragraph about the plausibility of our interpretation of oscillations in the ‘solution with oscillations’ section has been moved to the Discussion. We have made additional changes that we list below in response to individual points.

I leave below some comments and questions for the authors regarding clarity:

- I found the section "The problem of dimensionality and a solution with oscillations" particularly difficult to follow, and it is a crucial one. I had to read it multiple times to understand whether there was actually a new modeling/algorithmic solution compared to Belghazi et al. 2018. that would then be mapped to oscillations. From what I understand, besides opting for the Wasserstein-GAN/ALI objective, the difference is that here the authors allow for recurrent connections -- within a layer, only. So the essence of the proposed solution is to assume a hierarchical, layered model, and use layer-wise discriminators. The mapping to oscillations comes from the alternation between bottom-up and top-down (fast: say, for every data sample), actually as done by Belghazi et al. too. Is this correct?

Thanks, this is helpful feedback. We have added pseudocode and text to clarify what is different here with the Belghazi et al. approach. In summary, these difference are:
 - Discriminator architecture. We introduce one discriminator per layer. These are separate discriminators. Belghazi uses one large hierarchical discriminator for the whole net.
 - Layerwise recurrence.
 - Algorithm. The Belghazi algorithm does not ‘oscillate’ one layer at a time but rather all-the-way-up and then all-the-way-down. That is, we use each local discriminator to match p(z_i_|z_i+1_) with q(z_i_|z_i-1_), (where z_i+1_ is from the forward [q] distribution in both cases). Belghazi’s algorithm is entirely Wake/Sleep in the sense that they directly observe and adversarially align samples from the joint p(z) and q(z).

In responding to this point we discovered an errata in our own reference. We had originally written that “This strategy has been explored in the machine learning literature for strictly hierarchical architectures, as in this case one only needs to match the joint statistics over adjacent levels of the hierarchy of representations (Belghazi et al., 2018)” However, while Belghazi et al. indeed does analyze a hierarchical network, we found on a closer reading of Belghazi et al. that the architecture of the discriminator is not a series of separate discriminators that sees pairs of layers, as we had assumed, but rather a single discriminator taking as input the entire hierarchy. (See Table 4 in their Appendix; they hierarchically build a single main discriminator). Thus, our original reference was misleading.

The paragraph mentioning Belghazi et al. is now relocated to the end of this section, and is edited to read:

*“Algorithms which bear a close similarity to this have appeared in the machine learning literature. In the application of adversarial inference to hierarchical networks, the closest parallel work is Hierarchical Adversarially Learned Inference (HALI) \citep{belghazi2018hierarchical}. There are two main differences of the oscillatory algorithm with respect to HALI. First, HALI employs a single discriminator that observes the entire hierarchy of latent vectors, rather than the series of layerwise discriminators introduced here. Second, HALI only employs the generative model in a purely offline (Sleep-like) phase, whereas the oscillatory algorithm generates samples one layer down during the inference phase (Wake). HALI is thus best thought of as the application of the Adversarial Wake/Sleep algorithm to a hierarchical network, rather than an oscillatory algorithm that leverages the structure to factorize the discriminator.”*

- Pseudocode would help.

We have added pseudocode for both algorithms; the purely Wake/Sleep (which simply recapitulates the ALI algorithm) and the oscillatory algorithm.

- A clearer discussion comparing to the proposals of Deperrois et al. and Gershman would strengthen the paper. I'd also like to hear the author's speculation/thoughts on how biology would tackle the fragility issues of adversarial learning.

Thanks. We have added a paragraph to the discussion that contrasts our proposals with the proposals of Deperrois et al. and Gershman.

*“It is useful to contrast our algorithm with related hypotheses in which the brain learns adversarially \citep{gershman2019generative, deperrois2022learning}. Gershman similarly proposes that adversarial learning may underlie probabilistic inference and generative modeling in sensory cortex, which is the objective considered here and is identical to the ALI and BiGAN approach \citep{donahue2016adversarial, dumoulin2016adversarially}. Our biological proposal differs, however, in that we introduce cell types as discriminators whereas Gershman suggests that prefrontal cortex may play this role. We also consider the effect of adversarial fragility with dimension and as a solution propose purely local discriminators that leverage cortical oscillations. Deperrois et al. propose that it is the feedforward path of sensory cortex which plays the role of a discriminator of low-level inputs. Furthermore, in only aligning the activity distributions of the input areas, this proposal differs with ours and Gershman's, which aim to perform probabilistic inference over higher areas and thus align activity in all of sensory cortex. Deperrois et al. also propose a three-phase learning algorithm of wake, REM sleep, and NREM sleep, which serve to calculate an additional reconstruction error over inputs (wake phase) and a reconstruction error over top-level latents that are replayed by the hippocampus and then perturbed (in the NREM sleep phase). Our algorithm does not include these reconstruction errors or hippocampal replay. Overall, these central differences serve to highlight the importance of local discriminators with small input populations as well as preserving the high-level computational goal of probabilistic inference over generative models.”*

We have also brought up how our proposed experiments might separate these proposals in the section in the discussion about experiments.

With regard to the fragility of adversarial learning, it is difficult to add specific hypotheses about how the brain would stabilize learning because stability is not well understood in general. With that said, there is one interesting parallel between brains and stable GANs in that they both feature divisive normalization. We have added this to the Discussion paragraph on stability:

“*Finally, this fragility also makes it somewhat less plausible that evolution has found effective settings, though there possibly exist stabilizing features. For example, it is interesting that divisive normalization, which is a ubiquitous form of local recurrence in the cortex (Heeger et al. 1992), is also known to stabilize GAN training (Karras et al. 2017).*

- Typos should be screened for and corrected.

As for the experiments that the authors present, it seems to me that better understanding how recurrence affects the stability and practical feasibility of adversarial learning is one missing point. (As the authors remark, layerwise recurrence is not a standard feature of current machine learning models.) It might well be that recurrent models allow for more compact, efficient architectures (in loose analogy to e.g. deep equilibrium models, or energy-based models), but are otherwise harder to train. I understand that it's hard to answer such questions, but they would greatly add value to the paper. For instance, it would be interesting, and perhaps not too far-fetched, if recurrence would allow reducing layer size, which should not be too large for the sake of biological plausibility.

This is an interesting point. Indeed it would be very interesting to know what is the computational purpose and benefit of recurrence within the framework of adversarial learning. However, this is an exceedingly difficult question to answer and we doubt our ability to experimentally verify distinct improvements in machine learning benchmarks due to recurrence.

It is worth saying that our paper does not aim to justify recurrence with normative principles. Instead, it is to propose adversarial learning as a solution to recurrence’s existence, which we take for granted in the brain. Recurrence is the problem to solve (as in, “how might the brain perform probabilistic inference *despite* recurrence”) rather than a solution (as in, “how might recurrence improve inference and generative modeling”). The problem posed by recurrence is, of course, that it complicates conditional probabilities and prohibits many alternative algorithms for variational inference. One answer to “why adversarial learning in the brain?” is “Recurrence”.

Thus, if we introduced experiments in which we varied recurrence while holding layer size fixed, for example, it would seem to send the message that we believe there is some ‘optimal level of recurrence’. It is more true to our message to compare various algorithms on a network of fixed recurrence, which has been our approach thus far.

We are open to any suggestions as to how we might change this approach and central message.
